# Piperine improves ischemic brain injury by promoting the regulation of the AMPK/PGC-1α pathway by Apelin 13

**DOI:** 10.3389/fphar.2026.1746901

**Published:** 2026-01-22

**Authors:** Siyu Xi, Jiangbo Ma, Jing Yan, Yanzhong Li, Peng Zhang, Huiling Chen, Guangyu Yang, Xueyan Fu, Juan Liu, Yiwei Zhang

**Affiliations:** 1 School of Basic Medical Sciences, Ningxia Medical University, Yinchuan, China; 2 Key Laboratory of Ningxia Ethnomedicine Modernization, Minority of Education, Ningxia Medical University, Yinchuan, China; 3 School of Clinical Medicine, Ningxia Medical University, Yinchuan, China; 4 Wuhan Railway Vocational College of Technology, Wuhan, China; 5 School of Pharmacy, Ningxia Medical University, Yinchuan, China; 6 General Hospital of Ningxia Medical University, Yinchuan, Ningxia, China

**Keywords:** AMPK/PGC-1α pathway, Apelin 13, cerebral ischemia, mitochondrial biogenesis, piperine

## Abstract

**Background:**

Ischemic stroke (IS) persists as the second foremost cause of mortality and the primary cause of long-term disability globally, a burden largely attributable to a paucity of effective therapeutic strategies. Piperine (PIP) is a bioactive component of traditional Chinese medicine that has shown potential to reduce cell inflammation and pyroptosis. Recent studies indicate that mitochondrial biogenesis can improve ischemic stroke.

**Objective:**

In this study, we aimed to investigate the effect of PIP combined with Apelin 13 on mitochondrial biosynthesis in IS and determine its mechanism and whether PIP promotes Apelin 13.

**Methods:**

We used network pharmacology to screen chemical drugs for combination therapy for IS. Male Sprague–Dawley rats were utilized to induce a model of pMCAO, and primary cortical neuron cells were extracted to establish an oxygen–sugar deprivation–reperfusion model. To evaluate the changes in mitochondrial function of neuronal cells, we observed mitochondrial membrane potential via fluorescence microscopy, detected ROS levels by flow cytometry, and determined the ATP concentration by using a chemiluminescence multifunctional microplate reader. Western blot and qRT-PCR were used to detect the protein expression and mRNA content of Apelin 13 and the AMPK/PGC-1α pathway. In addition, the underlying mechanism of action of PIP promoting Apelin 13 in the regulation of the AMPK/PGC-1α pathway by using siRNA to reduce the content of Apelin 13 in primary cortical neurons was investigated.

**Results:**

The results of network pharmacology research indicated that Apelin 13 affects IS. PIP combined with Apelin 13 exerts neuroprotective effects against IS. The OGD/R group showed obvious mitochondrial functional damage, reduced mitochondrial membrane potential, increased reactive oxygen species level, and decreased ATP content compared with the Con group. Compared with the OGD/R group, the mitochondrial function detection and expression level of mitochondrial biogenesis-related factors in the PIP and Apelin 13 groups significantly improved, and the neuroprotective effect was more significant when the two were combined. Our *in vitro* and *in vivo* experiments revealed that, compared with the normal group, the mRNA and protein expression of Apelin 13 in the model group significantly decreased. Furthermore, the abundance of Apelin 13 in the PIP group substantially rose compared with that in the model group. When the expression of Apelin 13 was knocked down by si-Apelin 13, si-Apelin 13 effectively blocked the individual or even combined effects of PIP and Apelin 13.

**Conclusion:**

This study showed that PIP could promote Apelin 13 to activate mitochondrial biogenesis and decreased mitochondrial functional damage. The potential mechanism of activating mitochondrial biogenesis lies in the regulation of the AMPK/PGC-1α pathway. This study not only expands the understanding of the clinical application of PIP in the treatment of IS but also provides new insights into its internal mechanism.

## Graphical Abstract

**Figure:**
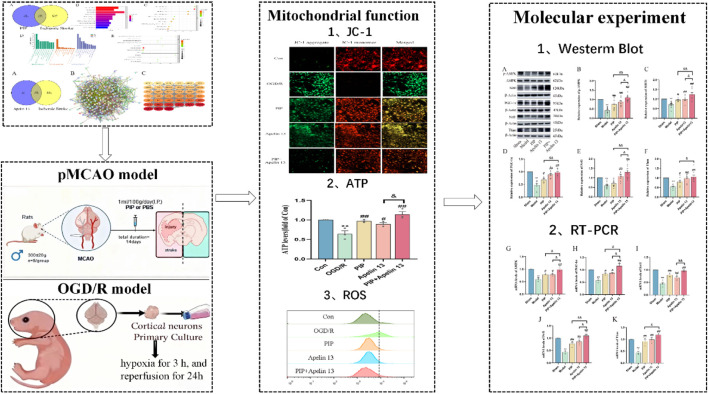

## Highlights

1. A systematic study was conducted on the therapeutic effects of PIP on ischemic stroke, focusing on a key aspect of ischemic brain injury: mitochondrial biogenesis.
2. Apelin 13 exerts neuroprotective effects against ischemic stroke by activating the AMPK/PGC-1α pathway in conjunction with PIP.
3. Discovery of the activating effect of PIP on the Apelin/AR system.

## 1 Introduction

Ischemic stroke (IS) is caused by the obstruction or constriction of the arteries that supply blood to the brain, resulting in insufficient blood flow and subsequent necrosis of brain tissue (Tuo et al., 2022). Stroke is a major contributor to high disability rates in the global burden of disease, and it has the second-highest mortality rate (Feigin et al., 2022), leading to a substantial disease burden and personal and socio-economic losses (Li et al., 2024). Recent advances in stroke treatment primarily involve vascular recanalization, but it can cause an immediate increase in blood sugar and blood oxygen levels, inducing secondary damage known as cerebral ischemia/reperfusion injury (Nour et al., 2012). Therefore, its pathogenesis should be urgently elucidated, and neuroprotective and restorative therapies must be explored to identify new treatment alternatives.

The multi-component nature of natural drugs can inhibit the damage cascade caused by ischemia at several targets and levels, presenting promising potential for the prevention and treatment of cerebral ischemic diseases (Zhu et al., 2022). Black pepper (*Piper nigrum* L.) is rich in sterols, terpenes, fatty acids, amides, and various other bioactive components, and it has broad prospects for development in the field of natural product research (Al-Khayri et al., 2022). Its core compound, piperine (PIP), possesses multiple effects such as antioxidant, anti-inflammatory, immune-regulating, and anti-apoptotic activities, and it can help prevent and treat neurological damage caused by cerebral ischemia in various ways (Haq et al., 2021).

The pathophysiological process of ischemic stroke is complex and involves multiple interacting signaling pathways, posing significant challenges to completely blocking it with conventional single treatments (Yu et al., 2025). Multi-target combined strategies coordinate the regulation of different pathological processes, enhancing efficacy while reducing drug dosage and side effects, showing significant advantages (Liu et al., 2021). Network pharmacology can systematically predict drug mechanisms and reveal principles of synergistic therapy by constructing “disease-target-drug” interaction networks (Wang et al., 2022; Wang et al., 2023). Therefore, this study intends to use network pharmacology methods to screen candidate chemical drugs for the combined treatment of ischemic stroke.

Network pharmacology results show that Apelin 13 has multiple overlapping targets associated with IS. Apelin was originally extracted from bovine gastric tissue and is the endogenous ligand of the G protein-coupled receptor APJ (Duan et al., 2019), which can regulate feeding and energy homeostasis in the hypothalamus (Mashaqi and Badr, 2019). Apelin can be degraded into four short active forms, namely, Apelin 12, Apelin 13, Apelin 17, and Apelin 36, among which Apelin 13 has the strongest biological activity (Xu et al., 2019). It exerts a wide range of biological activities through its receptor APJ, including antioxidant stress response, anti-apoptosis properties, and anti-inflammatory effects (Pouresmaeili-Babaki et al., 2018). One of the main signaling pathways of Apelin 13 relies on the interaction between G proteins associated with the APJ receptor and the protein AMPK (Kim et al., 2020). Research has shown that Apelin 13 treatment directly protects BMSCs from oxidative stress by activating AMPK-mediated mitophagy (Chen et al., 2021).

After cerebral ischemia, the blood supply to brain tissue is sharply reduced, causing metabolic dysfunction, intracellular calcium ion overload, mitochondrial membrane potential imbalance, abnormal ion gradient, ATP production interruption, and excessive ROS production (Chavda and Lu, 2023). Increased energy expenditure stimulates mitochondrial biogenesis, a mechanism aimed at enhancing cellular ATP production by generating more mitochondria (Herzig and Shaw, 2018). Any metabolic stress that interferes with ATP synthesis and disrupts energy homeostasis activates AMPK, which in turn reconfigures metabolism (e.g., regulating glycolysis and lipid synthesis) to restore energy balance. AMPK can directly phosphorylate PGC-1α, which can protect mitochondrial biosynthesis and function by promoting mitochondria-related gene expression and activity activation (Scarpulla, 2008). Tfam is a nuclear-encoded gene that facilitates communication between the nucleus and mitochondria and directly controlling mtDNA replication and transcription. Nrf1 is a pivotal upstream transcription factor that regulates mitochondrial biogenesis by synthesizing Tfam (Vozáriková et al., 2020). Therefore, we speculate that Apelin 13 may regulate the AMPK/PGC-1α/Nrf1/Tfam signaling axis and contribute to the modulation of mitochondrial biogenesis during PIP treatment following cerebral ischemia.

To investigate brain ischemic injury, we constructed *in vivo* and *in vitro* experimental models. The *in vivo* model utilized the rat permanent middle cerebral artery occlusion (pMCAO), whereas the *in vitro* model employed primary cortical neurons subjected to oxygen–glucose deprivation–reperfusion (OGD/R). This study aimed to evaluate the effect of PIP and Apelin 13 on neurological function recovery and mitochondrial biosynthesis after IS. In addition, the mechanism by which PIP confers protection against IS is revealed. The findings may provide a robust experimental standard for developing novel treatment methods for stroke.

## 2 Materials and methods

### 2.1 Network pharmacology: acquisition of compounds

Chem Draw was used to create a 2D structural diagram of PIP and Apelin 13. We saved their files in sdf format and exported the SMILES structure.

### 2.2 Drug target acquisition

First, the Swiss Target Database was used to retrieve results by inputting SMILES structural formulas and saving disease targets with probability >0 as drug targets. Second, the Pharmmapper database was used by uploading an sdf format file in the Submit Job section, changing the gene source to *Homo sapiens* for upload, filtering the results with Norm Fit >0.6, and transforming the targets into Gene Names via the UniProt database. Finally, the CTD database was used to enter the compound name, saving the results and removing duplicates to identify potential drug action targets.

### 2.3 Acquisition of disease targets

First, the GeneCards database was used to search for the keyword “Ischemic Stroke,” and disease targets were filtered with a relevance score greater than 10. Second, the DisGeNET database was used to input “Ischemic Stroke,” and all related diseases were selected for retrieval, yielding potential disease targets. Third, the OMIM database was searched with the keyword “Ischemic Stroke,” and gene names were saved. By deduplicating the above data, potential action targets of the disease were obtained.

### 2.4 Venn diagram drawing

Venny 2.1.0 was used to draw Venn diagrams by inputting the PIP, Apelin 13 targets, and disease targets to generate different drug target and disease response Venn diagrams.

### 2.5 KEGG and GO analyses

Core targets were used for the analysis to ensure the accuracy of the results. The core targets were entered into the DAVID database, and the gene source was set to *H. sapiens* for Kyoto Encyclopedia of Genes and Genomes (KEGG) analysis and Gene Ontology (GO) analysis. Relevant functional diagrams were drawn using bioinformatics tools.

### 2.6 PPI network construction and key target

Using the STRING database, we changed “Organisms” to *H. sapiens* to retrieve the intersection target PPI network diagram. Using the CentiScaPe 2.2 Menu plugin in Cytoscape to perform Degree, Closeness, and Betweenness analysis, key targets were identified.

### 2.7 Animals

In this experiment, Male Sprague–Dawley (SD) rats weighing 300 ± 20 g were acquired from the SPF animal facility of Ningxia Medical University. The rats were housed under standard specific pathogen-free (SPF) conditions with a controlled temperature of 22 °C ± 2 °C and a 12/12-h light/dark cycle. A 14-day acclimatization period was provided with free access to food and water to minimize stress-induced variability, ensuring the stability of animal physiological status before subsequent experimental procedures.

### 2.8 Cerebral ischemic injury (pMCAO) model

Given that middle cerebral artery occlusion (pMCAO) is a classic and clinically relevant model for simulating cerebral ischemia, we used this method to induce focal cerebral ischemic injury in rats. Briefly, animals were anesthetized by intraperitoneal injection of 7% chloral hydrate (0.5 mL per 100 g body weight) and fixed on their backs. Following exposure and careful isolation of the right CCA, ECA, and ICA, the proximal ECA and CCA were sutured, and blood flow in the distal ICA was arrested using microvascular clips. The sham operation group had a nylon wire insertion depth of 5 mm, and the remainder of the treatment was the same as that of the model group. The animals were kept warm with a thermal blanket at 37 °C before awakening and immediately transferred to room temperature after awakening. After the pMCAO model was established, the Longa neurological function score was assessed once the experimental animals regained consciousness. Only animals with a rating of 2 were incorporated into the experimental cohort, and all rats whose death and postoperative survival times did not meet the requirements of the collection time were excluded.

### 2.9 Lateral ventricular injection administration

To achieve targeted delivery of Apelin-13 to the central nervous system (the key site of cerebral ischemic injury), stereotactic lateral ventricular injection was performed after pMCAO model establishment. Rats were placed in the prone position to ensure their heads were centered and fixed in a brain stereotaxic apparatus. We shaved the scalp and disinfected it with iodine to fully expose the fontanelle. The sustained-release pump device was attached to the connecting rod of the brain stereotaxer. The angle of the needle tip was oriented vertically downwards. We ensured that the needle tip was aligned with the Y bifurcation of the fontanelle, which served as the origin of the brain stereotaxer with coordinates set to zero (X = 0, Y = 0). The coordinates of the injection site were X = −1.5, Y = −0.10. The rotation speed of the micro electric cranial drill was adjusted to align vertically with the coordinate point. The sustained-release pump needle was vertically inserted into a depth of 4.5 mm, fixed, and sealed with bone wax. Finnally,the skin was then sutured and disinfected.

### 2.10 Experimental design and dosing

The rats with a Zea-Longa score of 2 were randomly divided into four groups: model group, model + PIP group, model + Apelin 13 group and model + PIP + Apelin 13 group. The sham and model groups were subjected to daily gavage with 0.9% normal saline, serving as the vehicle-treated controls (1 m L/100 g). The PIP and PIP + Apelin 13 groups were given PIP (1 m L/100 g). The Apelin 13 and PIP + Apelin 13 groups were subjected to stereotactic injection after pMCAO. To align with the clinical window of opportunity for ischemic stroke treatment, all gavage administrations were initiated 24 h after ischemic injury and continued for 14 consecutive days to ensure sustained drug exposure.

### 2.11 Neuronal culture of neonatal rat primary cortical cells

Within 24 h of birth, SD suckling mice were disinfected with 75% ethanol and placed in a 10 cm Petri dish containing PBS solution placed on ice plates. The brain tissue was removed, and the bilateral cerebral cortex was isolated. The meninges and blood vessels were carefully removed by using miniature dissecting forceps, and they were transferred into a new Petri dish. The tissues were sectioned to a size of 1 mm^3^ and added with 0.25% trypsin at 37 °C for 20 min. After shaking for every 5 min, we added twice the volume of implant culture medium to terminate digestion. Following gentle aspiration with a straw several times, the supernatant was filtered through a 70-mesh screen into a new 15 mL conical tube. The precipitate was then discarded. The liquid in the centrifuge tube was centrifuged, the supernatant was discarded, and the precipitated cells were resuspended with an appropriate amount of implant solution and gently agitated 100 times. A cell counter was used to count the cells according to the experimental requirements for seeding into coated culture plates or dishes. After 24 h, the full amount of fluid was replaced with neuronal cell culture medium. The plate was gently agitated to remove cell debris before fluid replacement. After 48 h, 5 µM cytarabine was administered to purify the neurons, and the drug’s effect lasted 24 h.

### 2.12 Cell transfection

Primary cortical neuron cells seeded into 6-well plates were cultured for 7 days for cell transfection. Lipo3000 reagent and si-Apelin 13 were diluted in DMEM. The diluted Lipo3000 was evenly mixed with the si-Apelin 13 working solution and incubated at room temperature for 20 min. The Lipo3000-si-Apelin 13 mixture was introduced into the cell culture medium.

### 2.13 Cell grouping was established with the OGD/R model

The cellular population was categorized into seven distinct groups: Con group, OGD/R group, OGD/R + PIP group, OGD/R + Apelin 13 group, OGD/R + PIP + Apelin 13 group, si-NC (PIP drug and OGD/R treatment), and si-Apelin 13 (PIP drug and OGD/R treatment). PIP was diluted with Apelin 13 using medium to the desired concentration. The culture medium was changed to glucose-free medium and incubated in a hypoxic incubator at 37 °C with 5% CO_2_ and 1% O_2_ for 3 h.

### 2.14 Mitochondrial ROS detection

After drug intervention on primary cortical neuron cells inoculated in 6-well plates, we introduced 6 µL of DCFH-DA and 6 mL of medium to a test tube to prepare the working solution. About 1 mL was dispensed to a 6-well plate, which was incubated in an incubator at 37 °C. Subsequently, the cells were washed three times for on-machine testing.

### 2.15 Mitochondrial membrane potential detection

After the primary cortical neuron cells inoculated into a 6-well plate were intervened with drugs, the cell culture solution was removed, washed once with PBS, added with 1 mL of diluted JC-1 staining working solution, mixed well, and incubated for 20 min. Thereafter, the liquid was discarded, and the cells were washed twice. Finally, the 6-well plate was observed using a fluorescence microscope.

### 2.16 RT-PCR

About 1 mL of lysate RZ was added per 50 mg of tissue or 500 µL of lysate was incorporated per 1 × 10^6^ cells. The homogenate was thoroughly mixed and allowed to stand at room temperature for 10 min to achieve complete lysis. Subsequently, it was placed in a centrifuge at 4 °C and 12,000 rpm for 5 min to remove the supernatant. We added 200 µL of chloroform to every 1 mL of lysate, mixed them thoroughly, and centrifuged them to obtain the supernatant. The upper layer of the aqueous phase was extracted. After adding 0.5 times its volume of anhydrous ethanol, we mixed the sample gently, and centrifuged it to discard the waste liquid in the collection tube. After drying, we added DEPC water to dissolve the RNA. We then used the ACScript RT PreMix Kit (RK20429, Abclone, China) for reverse transcription into complementary DNA (cDNA). The sample was then loaded according to the system and placed on a PCR instrument for reaction. The primer sequences for gene detection are listed in Table 1.

**TABLE 1:** Primer sequences for RT-PCR.

Primer name	Primer sequences (5′-3′)
R-GAPDH-S	CTG​GAG​AAA​CCT​GCC​AAG​TAT​G
R-GAPDH-A	GGT​GGA​AGA​ATG​GGA​GTT​GCT
R-AMPK-S	ATA​GCC​GAC​TTC​GGT​CTT​TCA
R-AMPK-A	CTT​CAG​GGC​CTG​CGT​ACA​ATC
R-Sirt1-S	TGA​CCT​CCT​CAT​TGT​TAT​TGG​G
R-Sirt1-A	GGC​ATA​CTC​GCC​ACC​TAA​CCT
R- PGC-1α-S	AAT​CAA​GCC​ACT​ACA​GAC​ACC​G
R- PGC-1α-A	ATT​GCT​TTC​TGC​TTC​TGC​CTC​T
R-Nrf1-S	TCA​TGG​ACC​CAA​GCA​TTA​CG
R-Nrf1-A	AAC​CTG​GAT​GAG​CGA​CAC​G
R-Tfam-S	GGC​AGA​AAC​GCC​TAA​AGA​AGA​A
R-Tfam-A	AAC​TTC​AGC​CAT​TTG​CTC​TTC
R-Aplnr-S	CCT​ACC​GGG​AGT​TTG​ACT​GG
R-Aplnr-A	GCA​GCC​TTA​GTC​GAG​CGT​TA
R-Apln-S	CTC​TCC​TTG​ACT​GCC​GTG​TGT
R-Apln-A	GTA​GCG​CAT​GTT​GCC​TTC​TTC​T

### 2.17 Western blot

Tissue and cell suspension were collected; PMSF cell lysate was added; homogenization was followed by centrifugation to remove the supernatant; and 2× SDS loading buffer was added and heated in a boiling water bath, cooled, dispensed, and stored at −20 °C. Electrophoresis was conducted as follows: electrophoresis at 80 V for concentrated glue for 25 min, followed by separation at 120 V until bromophenol migrated to the bottom of the gel. Thereafter, electrophoresis was terminated, and film transfer was carried out. The power supply was activated, and 200 mA was transmitted for 2 h. After the transfer of the film was completed, the PVDF film was removed, placed in the sealing solution, and sealed on a horizontal shaker at room temperature. The blocking solution was washed with TBST, and the PVDF membrane was immersed in the primary antibody dilution containing the primary antibody and agitated on the horizontal shaker overnight. The PVDF membrane was then washed with TBST three times and added with the secondary antibody for incubation. The film was washed with PBST three times. Lastly, it was applied to the PVDF membrane for exposure, development, and analysis of the protein banding results of interest with ImageJ software.

### 2.18 Statistical analysis

All data were presented as mean ± SEM, based on at least three independent experiments. Comparisons between groups were performed using one-way ANOVA. *P < 0.05* was considered statistically significant.

## 3 Results

### 3.1 Venn diagram of PIP and IS target

**FIGURE 1:**
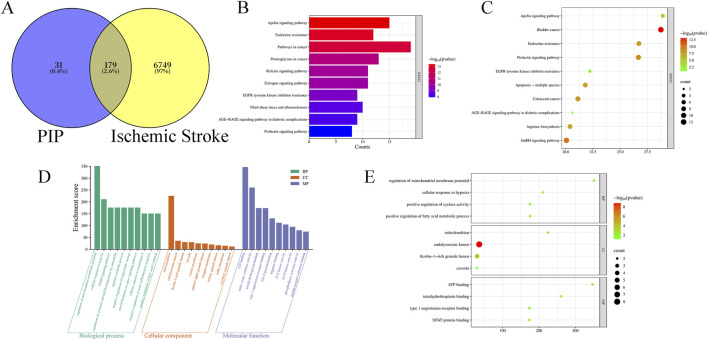
**(A)** Venny diagram of PIP targets and disease targets. **(B)** PIP KEGG enrichment bar Chart. **(C)** PIP KEGG enrichment Bubble Chart. **(D)** PIP GO intersection target Rectangular Chart. **(E)** PIP GO intersection target Bubble Chart.

A total of 6,928 IS targets were obtained through database searches. PIP had 210 targets, of which 179 were overlapping (Figure 1A).

### 3.2 KEGG analysis

Using DAVID, a total of 132 pathways related to PIP and IS were identified. The top ten pathways were selected and ranked according to “Fold Enrichment,” indicating the effectiveness of the Apelin pathway (Figures 1B,C).

### 3.3 GO enrichment analysis

GO analysis was performed using the DAVID database. The results showed that PIP comprised 264 biological processes (BP), 33 cellular components (CC), and 77 molecular functions (MF). With *P < 0.001* as the screening criterion, the results showed that PIP BP was mainly concentrated in cellular response to hypoxia and regulation of mitochondrial membrane potential, PIP CC was mainly concentrated in the mitochondrion, and MF was mainly concentrated in ATP binding (Figures 1D,E).

### 3.4 Venn diagram of Apelin 13 and IS target

**FIGURE 2:**
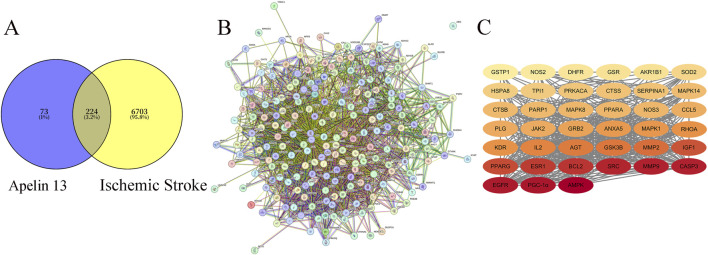
**(A)** Venny Diagram of Apelin 13 Targets and Disease Targets. **(B)** STRING Apelin 13- Ischemic stroke -Target Protein-Protein Interaction (PPI) Network. **(C)** Diagram of Core Targets of Apelin 13 (Note: The darker the color, the higher its Degree value).

A total of 6927 IS targets were obtained *via* database retrieval. The drug target for Apelin 13 was 297, with 224 intersecting targets (Figure 2A).

### 3.5 PPI network mapping and core target prediction of Apelin 13

The intersection target PPI network mapping for Apelin 13 yielded a table (Figure 2B; Table 2), from which the core target count for Apelin 13 was obtained. The relevant diagrams are shown in Figure 2C.

**TABLE 2:** STRING drug-disease-target network.

Drug	Point	Side
Apelin 13	39	447

“points” refer to the number of intersection targets, and “edges” represent the interaction relationships between the targets. The more “edges” there are, the more complex the interactions.

### 3.6 PIP collaborates with Apelin 13 to improve mitochondrial function

**FIGURE 3:**
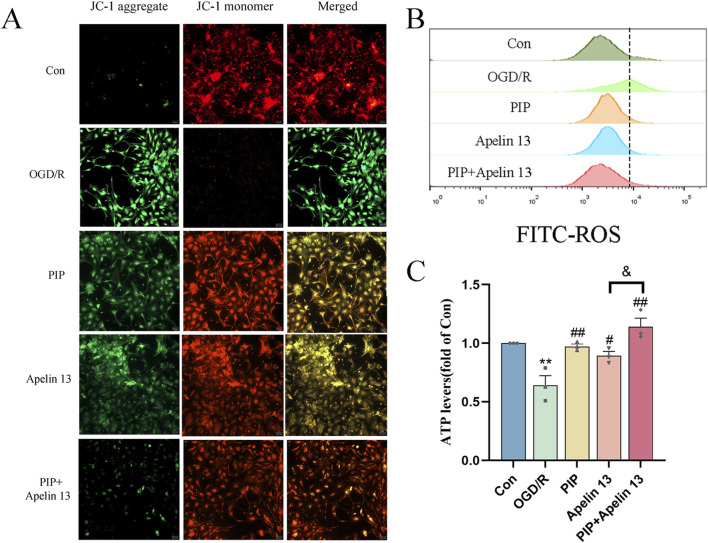
PIP synergizes with Apelin 13 to improve mitochondrial function. **(A)** JC-1 fluorescence was observed by fluorescence microscope to detect MMP, acollapse in mitochondrial membrane potential is indicated by an increase in the number of cells shifting from red to green fuorescence (Bar = 50 um). **(B)** ROS generation was determined with DCFH-DA staining and analyzed by Flow Jo. **(C)** ATP levels were quantified in primary cortical neuron cells. Compared with the Con group, **P < 0.01, compared with the OGD/R group, ^*#*^ *P < 0.05* ^*##*^ *P < 0.01*, compared with the PIP + Apelin 13 group, ^*&*^ *P < 0.05*, (n ≥ 3).

To confirm the protective effect of mitochondrial function damage and PIP and Apelin 13 in IS, we detected primary cortical neuron cells treated with DCFH-DA by flow cytometry (Figure 3A). OGD/R led to an increase in intracellular ROS levels compared with normal cells after OGD/R treatment. Compared with the model cells, the ROS level decreased after PIP and Apelin 13 treatment, indicating that PIP and Apelin 13 could inhibit the increase in ROS levels in mitochondria induced by OGD/R.

ATP is a basic source of energy for brain tissue activity and is the main substance that maintains cell function. To explore whether PIP and Apelin 13 have a positive effect on OGD/R-induced mitochondrial damage, we detected the ATP level via chemiluminescence (Figure 3B). We found that OGD/R significantly reduced intracellular ATP levels, which demonstrated a statistically significant deviation from those of the control cohort (*P < 0.01*), indicating that mitochondrial respiratory function was inhibited. In the PIP and Apelin 13 treatment groups, the intracellular ATP levels exhibited a marked elevation, and the ATP content increased significantly when PIP was combined with Apelin 13. Thus, PIP and Apelin 13 could restore mitochondrial respiratory function to a certain extent, and the two had a synergistic effect.

A widespread application for assessing mitochondrial membrane potential (MMP) is the use of the fluorescent probe JC-1. Its fluorescence properties change with the change in MMP: when the MMP is high, JC-1 aggregates and assembles into polymers in the mitochondrial matrix, emitting red fluorescence. When the membrane potential decreases, JC-1 exists in the form of a monomer and emits green fluorescence. As shown in Figure 3C, the green fluorescence of the Con group was weak and the red fluorescence was strong, indicating that most of the JC-1 was ingested into the mitochondria to form polymers, and the MMP was normal. After OGD/R treatment, the green fluorescence was significantly enhanced, and the red fluorescence was significantly weakened, indicating that OGD/R led to a decline in the MMP in cells. After the intervention of PIP and Apelin 13, the green fluorescence was markedly weakened relative to the OGD/R group, and the red fluorescence was significantly enhanced, indicating that PIP and Apelin13 could protect the mitochondria against OGD/R-impaired function. The relative proportion of red and green fluorescence after PIP was combined with Apelin 13 was higher than that of the two, indicating that the two had a synergistic effect on improving mitochondrial function.

### 3.7 PIP synergistically regulates the AMPK/PGC-1α signaling pathway with Apelin 13 to exert a brain protective effect

**FIGURE 4:**
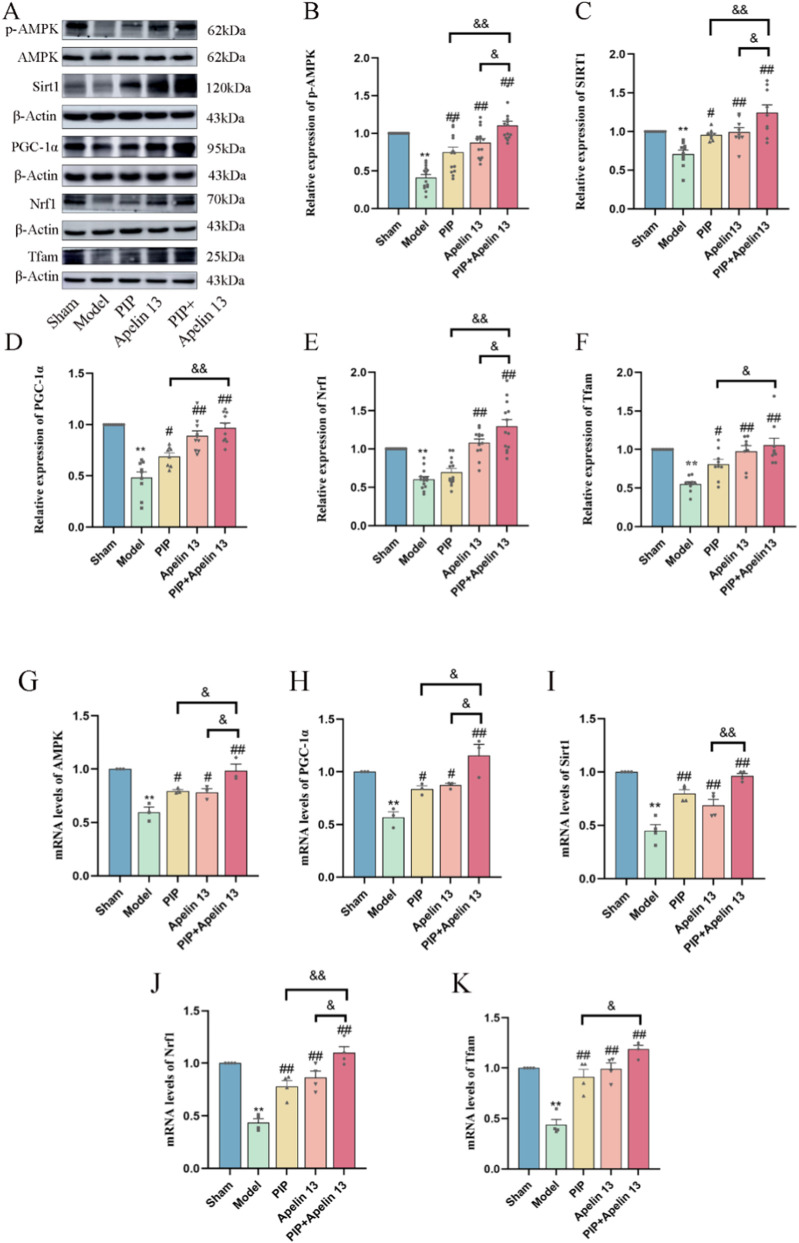
PIP cooperates with Apelin 13 to regulate AMPK/PGC-1α signaling pathway to play a protective role in the brain. **(A–F)** Western blot analysis of p-AMPK/AMPK, SIRT1, PGC-1α, Nrf1, Tfam protein expression level in brain tissue. **(G–K)** The mRNA expression levels of AMPK, SIRT1, PGC-1α, Nrf1, Tfam gene in brain tissue. Compared to the Sham group, *P < 0.05**P < 0.01, Compared to the Model group, ^*#*^ *P < 0.05* ^*##*^ *P < 0.01*, Compared to the PIP + Apelin 13 group, ^*&*^ *P < 0.05* ^*&&*^ *P < 0.01* (n ≥ 3).

Given that PIP has a neuroprotective effect on IS, and Apelin 13 is a brain-protective factor, we hypothesized that PIP synergizes with Apelin 13 to improve brain injury. Therefore, we performed Western blot and qRT-PCR experiments to observe the activation effect of PIP and Apelin 13 on the AMPK/PGC-1α/Nrf1 pathway proteins in brain cells. Compared with the sham group, the protein levels of pAMPK/AMPK, SIRT, PGC-1α, Nrf1, and Tfam in the model group were significantly reduced (*P < 0.01*). Compared with the model group, the levels of pAMPK/AMPK, PGC-1α, Nrf1, and Tfam proteins in the PIP and Apelin 13 groups increased (*P < 0.05*), but the increase in the PIP Apelin 13 group was more obvious (Figures 4A–F).

The results of RT-PCR (Figures 4G–K) showed that the gene expression levels in the model group exhibited a significant decrease relative to the sham group (*P < 0.01*). Compared with the model group, the expression levels of the AMPK, PGC-1α (*P < 0.05*), SIRT, and Tfam (*P < 0.01*) genes in the PIP group significantly increased, but the expression of the Nrf1 genes did not change significantly. The gene expression levels of AMPK, PGC-1α (*P < 0.05*), SIRT, Nrf1, and Tfam (*P < 0.01*) in the Apelin 13 group also increased, and the expression levels of AMPK, SIRT, PGC-1α, Nrf1, and Tfam in the PIP Apelin 13 group significantly increased (*P < 0.05*) compared with those in the PIP and Apelin 13 groups. The outcomes of Western blot and qRT-PCR analyses demonstrated that the coadministration of PIP and Apelin 13 enhanced the neuroprotective effect.

### 3.8 PIP promotes the expression of Apelin 13

**FIGURE 5:**
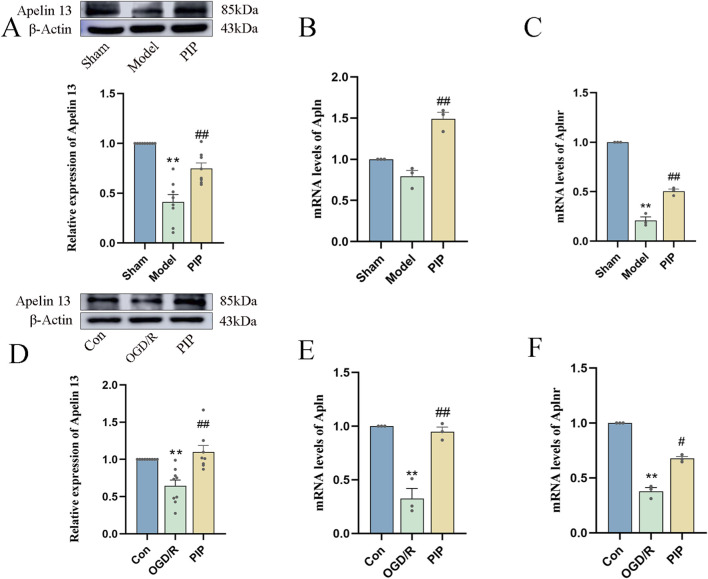
PIP promotes the expression of Apelin 13 gene and protein in brain tissue and cells. **(A)** Effects of PIP on the protein levels of Apelin 13 in the brain tissues; **(B)** Effects of PIP on the mRNA levels of Apln in the brain tissues; **(C)** Effects of PIP on the mRNA levels of Aplnr in the brain tissue; **(D)** Effects of PIP on the protein levels of Apelin 13 in primary neuronal cells; **(E)** Effects of PIP on the mRNA levels of Apln in primary neuronal cells; **(F)** Effects of PIP on the mRNA levels of Aplnr in primary neuronal cells; Compared to the Sham group, **P < 0.01, Compared to the Model group,^*#*^ *P < 0.05* ^*##*^ *P < 0.01*, (n ≥ 3).

To determine whether PIP promotes Apelin 13 in brain tissue or cells, we employed Western blot and RT-PCR to detect the levels of Apelin 13 protein and its receptor Aplnr gene in brain tissue and Apelin 13 protein. As shown in Figures 5A,D, MCAO and OGD/R significantly decreased the expression level of Apelin 13 protein in brain tissue compared with the normal group (*P < 0.01*), whereas the expression level of Apelin 13 protein in brain tissue and primary neuron cells in the PIP group demonstrated a significant upregulation relative to the model cohort (*P < 0.01*). RT-PCR results showed (Figures 5B,C,E,F) that Apln and Aplnr gene expression levels were significantly inhibited by OGD/R (*P < 0.01*), whereas PIP promoted Apln and Aplnr gene expression (*P < 0.01*). These results suggested that PIP could activate the expression of Apelin 13 in brain cells and may play a therapeutic role by regulating Apelin 13.

### 3.9 PIP exerts neuroprotective effects by promoting the regulation of the AMPK/PGC-1α signaling pathway via Apelin 13

**FIGURE 6:**
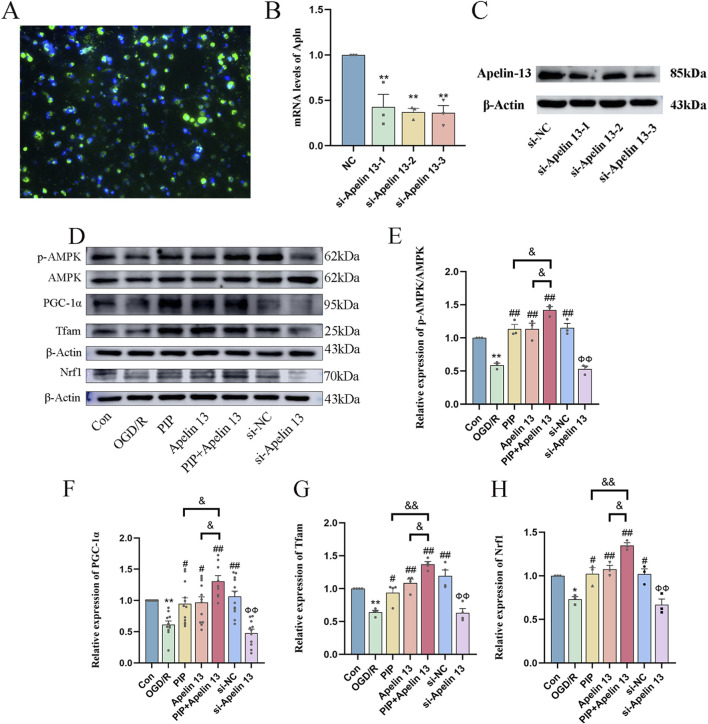
PIP exerts neuroprotective effects by promoting the regulation of AMPK/PGC-1α signaling pathway by Apelin 13. **(A)** Fluorescence staining image of si-Apelin 13 (bar = 50 μm); **(B,C)** The quantitative analysis of gene silencing level of si-Apelin 13 and western bands of si-Apelin 13; **(D–H)** Western blot analysis of p-AMPK/AMPK, PGC-1α, Tfam, Nrf1 protein expression level in primary neurons. Compared to the Con group, *P < 0.05**P < 0.01, Compared to the OGD/R group, ^*#*^ *P < 0.05* ^*##*^ *P < 0.01*, Compared to the PIP + Apelin 13 group, ^*&*^ *P < 0.05* ^*&&*^ *P < 0.01*, Compared to the si-NC group, ^*Ф*^ ^*Ф*^ *P < 0.01* (n ≥ 3).

To further verify whether PIP regulates AMPK/PGC-1α by promoting Apelin 13, we used siRNA to silence Apelin 13 in primary cortical neuron cells (Figures 6A–C) to detect its role in mitochondrial biosynthesis. Western blot was used to detect the protein expression of the AMPK/PGC-1α pathway factor. Compared with the Con group, the protein expression levels of AMPK, PGC-1α, Tfam, and Nrf1 in the OGD/R group decreased. Compared with the OGD/R group, the protein expression levels of AMPK, PGC-1α, Tfam, and Nrf1 in the PIP group, Apelin 13 group, PIP Apelin 13 group, and si-NC group significantly increased, and the synergistic effect of PIP and Apelin 13 was particularly evident. Compared with the si-NC group, the protein expression levels of pAMPK, PGC-1α, Tfam, and Nrf1 factors in the si-Apelin 13 group were significantly lower (^*Ф*^ ^*Ф*^ *P < 0.01*).

## 4 Discussion

IS is a leading global cause of disability and death, posing severe economic burdens on families and society (Kuriakose and Xiao, 2020). Tissue plasminogen activator (tPA), the only FDA-approved thrombolytic drug for IS, is restricted to a 4.5-h post-stroke window (Fukuta et al., 2017). Additionally, using tPA carries other associated risks, such as allergic reactions and systemic bleeding (Chapman et al., 2014). Thus, there is an urgent need for effective therapeutic strategies. Natural medicines have unique advantages in the protection of ischemic brain injury because of their multi-component and multi-target properties (Zhu et al., 2021). Our previous studies showed that PIP extract demonstrates significant neuroprotection in in vivo and *in vitro* models of IS. Our investigation revealed that PIP confers neuroprotection against ischemic brain injury via suppression of the caspase-1-dependent pyroptosis signaling cascade (Lu et al., 2025). In addition, tetrahydropiperine can inhibit excessive autophagy by activating the PI3K/AKT/mTOR pathway, thereby promoting the repair of ischemic penguilacytic zone neurons after cerebral infarction (Ren et al., 2024). Given the complexity of the pathological mechanisms of IS, the combined effect of multiple drugs holds greater promise. And network pharmacology can provide a comprehensive theoretical foundation and methodological support for it (Jiang et al., 2022). The results from network pharmacology, JC-1, ROS, and ATP further confirmed that PIP and Apelin 13 can induce mitochondrial biogenesis, thereby improving mitochondrial dysfunction. *In vivo* and *in vitro* Western blot and RT-PCR results showed that PIP had a promoting effect on Apelin 13. In addition, siRNA was used to knock down the content of Apelin 13 in primary cortical neurons, and si-Apelin 13 weakened the promotion effect of PIP and Apelin 13 on mitochondrial biogenesis-related factors AMPK/PGC-1α. These data suggested that PIP may improve ischemic brain injury by promoting Apelin 13 and modulating the AMPK/PGC-1α pathway.

When the brain is low in oxygen and glucose levels, it becomes susceptible to sudden cerebral blood flow disruptions, which trigger substantial reactive ROS. Excess ROS attack the mitochondrial membrane, causing increased membrane permeability and impaired structural integrity and inducing the opening of mitochondrial permeability transition pore (Sanderson et al., 2013; Daskalaki and Tavernarakis, 2020). Impaired mitochondrial function reduces ATP production, failing to meet the body’s repair demands. Consequently, cells in the ischemic penumbra cannot self-repair, leading to expanded infarct regions and aggravated abnormal brain function (Woodruff et al., 2011). Similarly, our GO enrichment analysis results also indicated that the role of PIP was mainly concentrated in these processes. We found that the importance of PIP and Apelin 13 lies in their ability to seem to break this cascade. They reduce ROS levels, protect mitochondrial integrity, and restore ATP levels, thereby demonstrating a protective mechanism that directly resists ischemic injury.

Apelin 13 and its binding receptor APJ are widely expressed in the central nervous system, especially in the hypothalamus, hippocampus, cortex, and substantia nigra (Duan et al., 2019). As a neuropeptide, various roles of Apelin 13 in regulating the occurrence and development of IS have been reported. Studies have shown that Apelin 13 exerts a neuroprotective effect on cerebral ischemia–reperfusion injury *in vivo* and *in vitro* by inhibiting cell apoptosis and excessive autophagy (Shao et al., 2021). However, Apelin 13 is a peptide substance, so it has difficulty in crossing the blood–brain barrier. KEGG enrichment analysis results showed that one of the pathways associated with IS in PIP is the Apelin signaling pathway. Thus, we speculated that PIP can activate Apelin 13 and then effectively regulate the Apelin system to exert a brain-protective effect. To verify whether this hypothesis is feasible, we performed RT-PCR and Western blot to detect the gene expression of Apelin 13 and its ligands and the protein expression of Apelin 13 in pMCAO rats and primary cortical neurons. We found that the expression level of Apelin 13 significantly increased after treatment with PIP drugs. These results indicated that PIP could promote the expression of Apelin 13, consistent with our network pharmacology results.

Mitochondrial biogenesis is the process by which cells generate new mitochondria from pre-existing ones to enhance mitochondrial quantity and function (Jamwal et al., 2021). with the AMPK-PGC-1α axis as its main regulatory pathway (Kleinschnitz et al., 2010). AMPK directly enhances the expression of PGC-1α, migrating from the cytoplasm to the nucleus, activating various transcription factors including Nrf1 and Nrf2, and promoting regions of mitochondrial transcription factor A (Tfam) (Aquilano et al., 2010; Jornayvaz and Shulman, 2010; Gleyzer et al., 2005). In addition, Apelin 13 can upregulate AMPK phosphorylation levels in cerebral ischemic injury (Duan et al., 2019). Subsequently, following *in vivo* modeling, our analysis indicated a decrease in the expression of related transcription factors in the mitochondria. After the intervention of PIP and Apelin 13, the phosphorylation of AMPK was elevated, accompanied with a markedly upregulated secretion of PGC-1α, Nrf1, and Tfam proteins, which was consistent with related research (Kong et al., 2021). These results further support the view that the combination of PIP and Apelin 13 can promote the generation of new mitochondria in brain tissue, while sufficient energy supply helps the recovery and reconstruction of damaged neurons in the injured cerebral cortex regions.

After finding that PIP can promote the expression of Apelin 13, and both can promote a significant increase in mitochondrial transcription factors related to the AMPK/PGC-1α pathway, combined with the results of previous PCR analysis, we speculated that Apelin 13 is a key target. Subsequently, we transfected si-Apelin 13 into primary cortical neurons to knock down the content of Apelin 13 in cells to determine whether this factor serves as a mediator. The results showed that si-Apelin 13 effectively blocked the individual and combined effects of PIP and Apelin 13. This was also consistent with our previous observations, which showed that Apelin 13 mediated the recovery of neurological damage by the AMPK/PGC-1α pathway and was a potential target for the management of IS. However, one limitation of this study is that it did not observe damage to mitochondrial function *in vivo*. To address this limitation, we will continue to conduct additional experiments in this area in subsequent studies.

## 5 Conclusion

In conclusion, this study demonstrated that PIP can promote Apelin 13 to activate mitochondrial biogenesis and reduce mitochondrial functional damage. Its role in activating mitochondrial biogenesis may be related to the regulation of the AMPK/PGC-1α pathway. The findings of this study elucidate novel perspectives on the therapeutic application of PIP for cerebral ischemia and clarify its molecular mechanisms.

## Data Availability

The datasets presented in this study can be found in online repositories. The names of the repository/repositories and accession number(s) can be found in the article/Supplementary Material.
