# Supplementary figures and images for: Piperine improves ischemic brain injury by promoting the regulation of the AMPK/PGC-1α pathway by Apelin 13
Source: Front Pharmacol. 2026 Jan 22;17:1746901. doi: 10.3389/fphar.2026.1746901 (PMC12872509; doi:10.3389/fphar.2026.1746901)

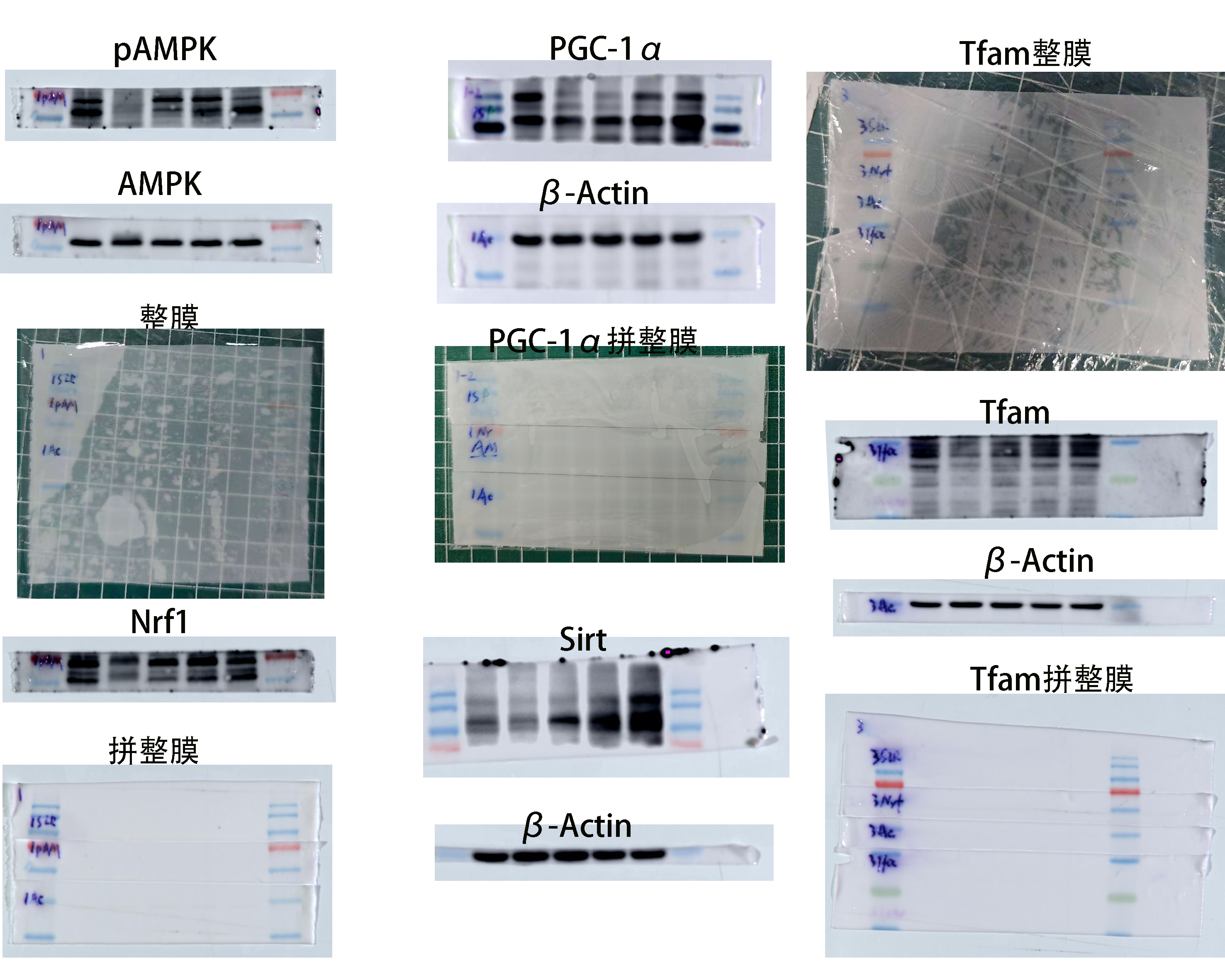

Supplement: Supplementary file 1 [file Image3.tif]

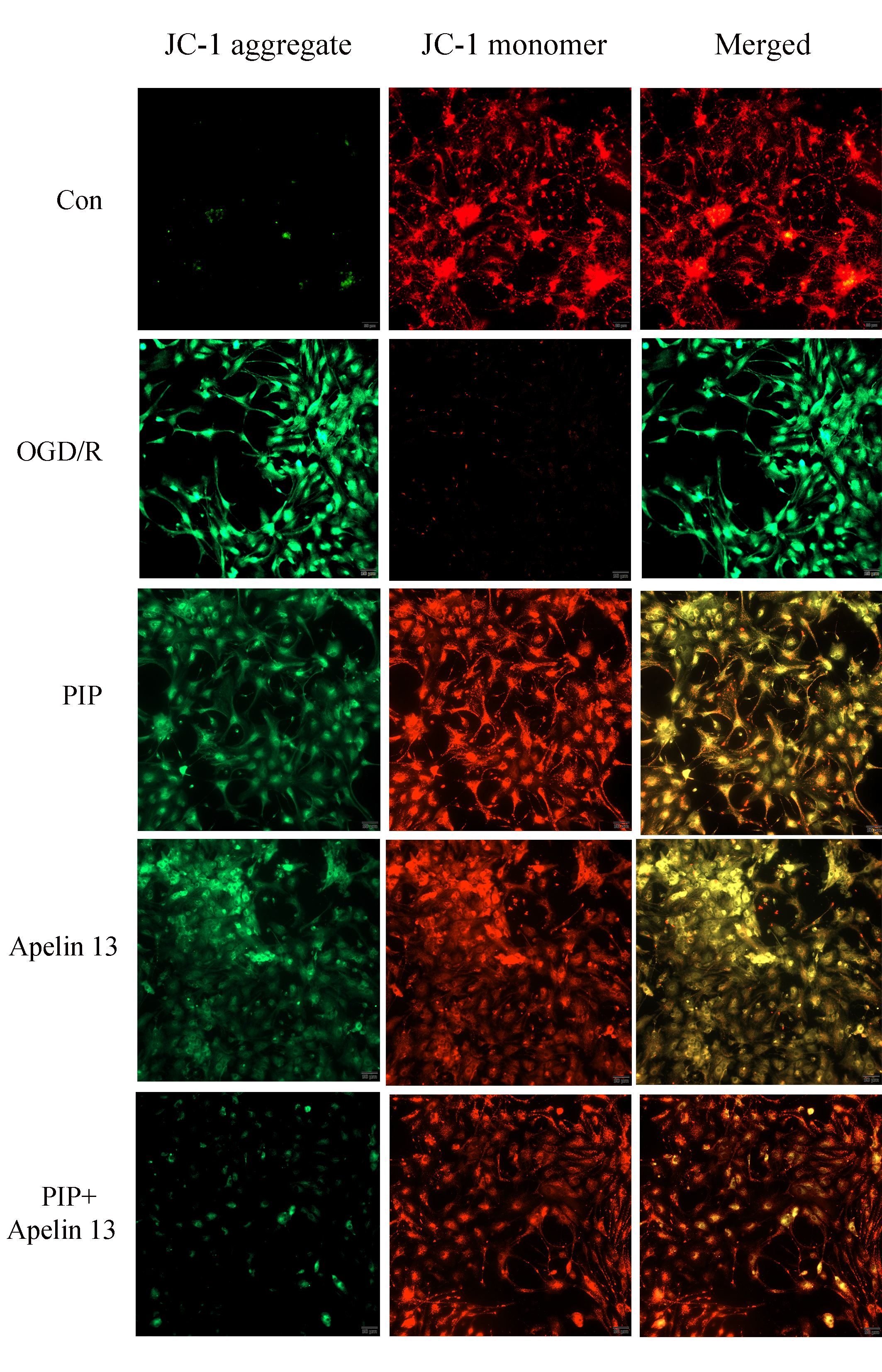

Supplement: Supplementary file 2 [file Image4.jpeg]

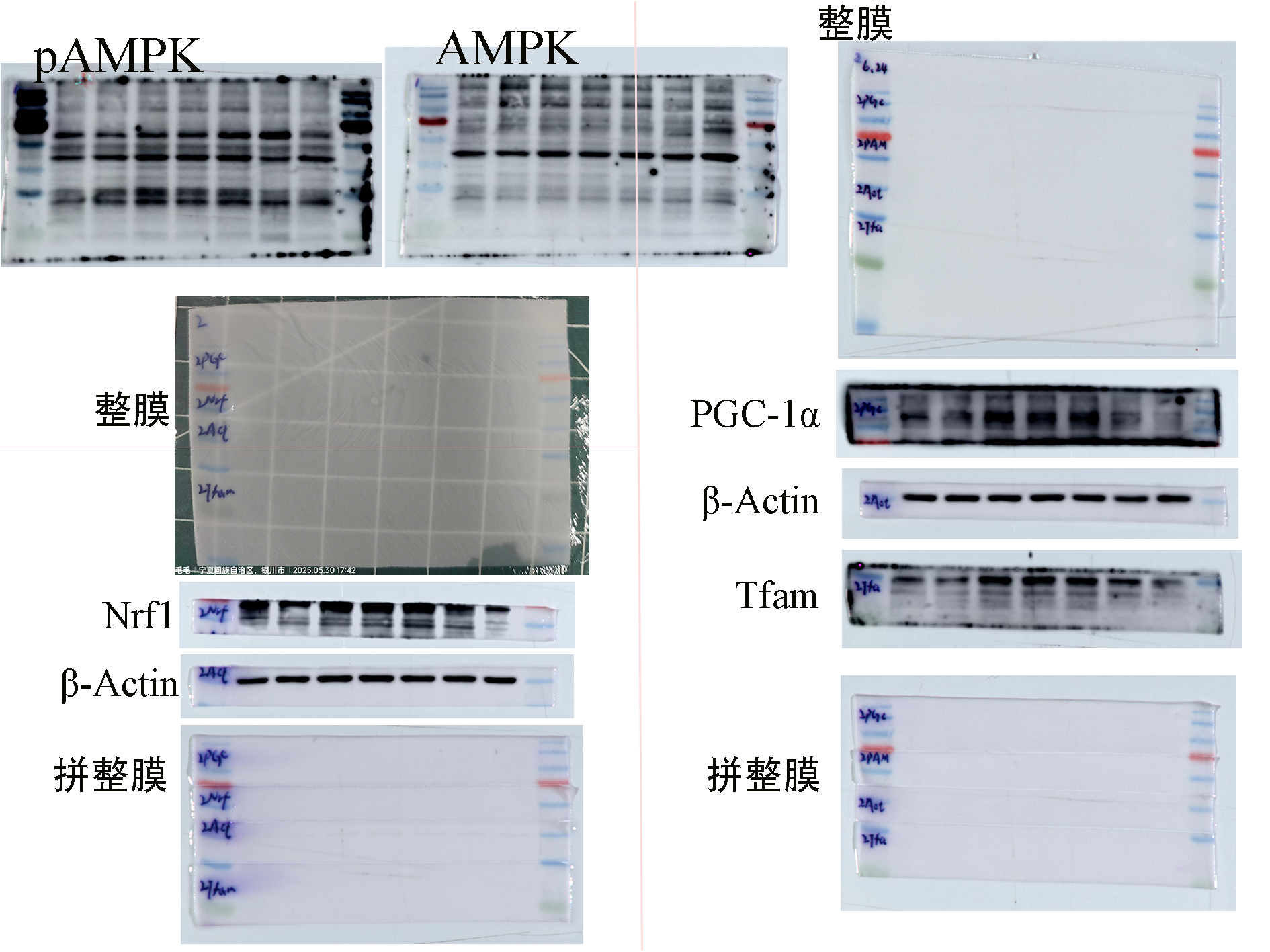

Supplement: Supplementary file 3 [file Image2.tif]

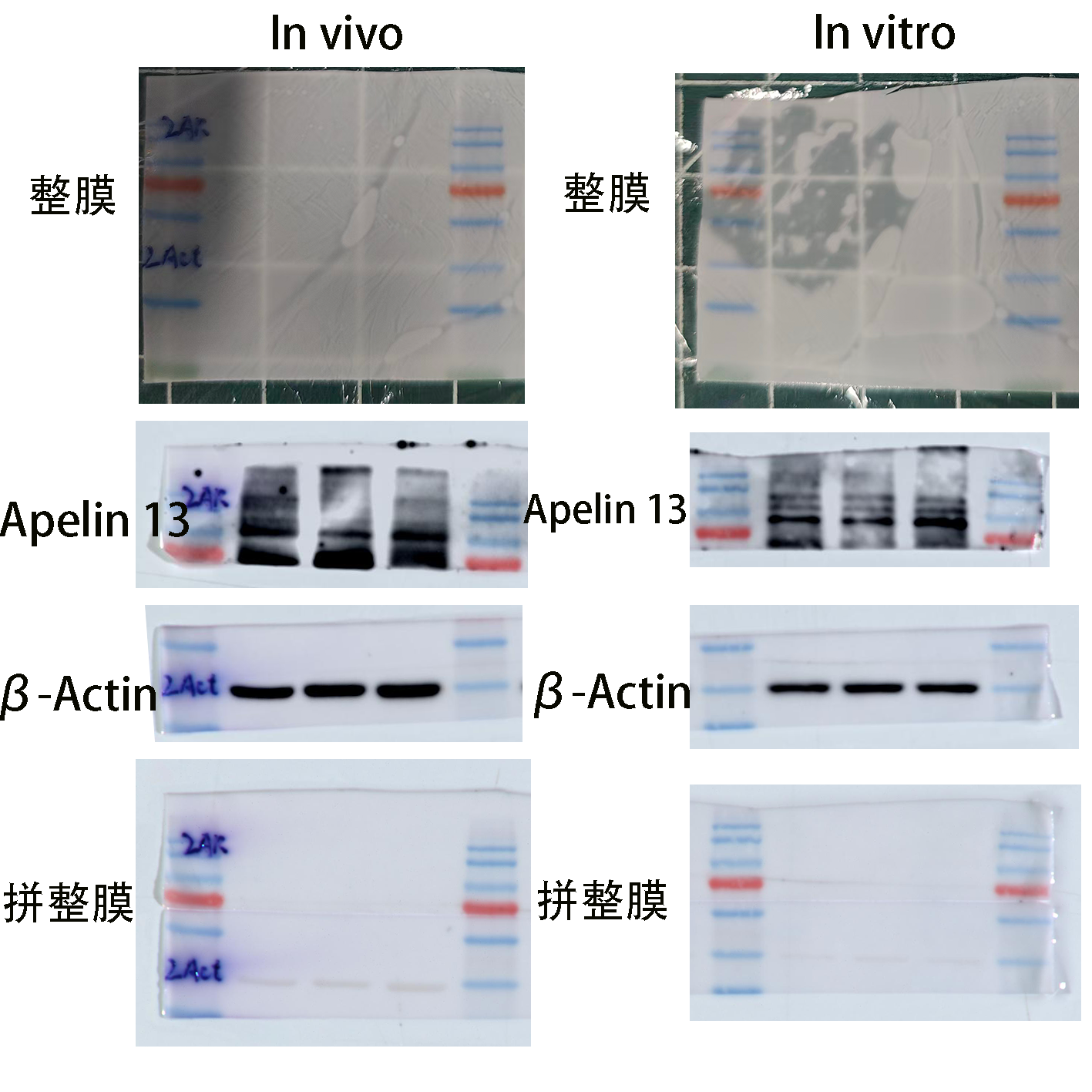

Supplement: Supplementary file 4 [file Image1.tif]
